# Supplementary material for: The consequences of strategic prioritization in working memory
Source: Psychon Bull Rev. 2026 Jun 2;33(5):167. doi: 10.3758/s13423-026-02922-7 (PMC13661736; doi:10.3758/s13423-026-02922-7)
Supplement: Supplementary file 1 [file 13423_2026_2922_MOESM1_ESM.docx]

**Supplementary materials**

**1. Experiment 1: Sample size and Bayesian sequential hypothesis testing**

As preregistered (see <https://osf.io/ac5vw>), we used Bayesian sequential hypothesis testing. In particular, we planned to start with a first batch of 120 participants (i.e., 30 participants in each experimental group: Test-relevance, Reward, Refreshing, and Test-relevance&Reward; with half of the participants per group recruited in Geneva and the other half in Austin). We planned to analyze the data of this first batch to assess the evidence in the data for or against the interaction of interest (the interaction between Priority Type and Priority Status). If the Bayes factor for or against the interaction of interest would be lower than 10, a second batch of 120 participants would be tested (i.e., again 30 participants in each experimental group: Test-relevance, Reward, Refreshing, and Test-relevance&Reward; with half of the participants per group recruited in Geneva and the other half in Austin). Thus, testing would either stop after the first batch of 120 participants because the Bayes factor of interest reached 10, resulting in a final sample size of 120 participants, or stop after the second batch of 120 participants because the Bayes factor of interest did not reach 10 after the first batch, resulting in a final sample size of 240 participants.

In the first batch, we collected data of 122 participants in total, resulting in 104 usable datasets after performing preregistered performance-based exclusions (see main text). The best model of these data was the Priority Status-only model, and this model was 6.36 times better than the full model including the interaction of interest. As this Bayes factor was lower than 10, we collected a second batch of data. In this second batch, we collected data of 129 participants in total. As such, we collected data of 251 participants in total for Experiment 1.

**2. Experiment 2: Sample size and Bayesian sequential hypothesis testing**

As preregistered (see <https://osf.io/92ufg>), we used Bayesian sequential hypothesis testing. Like in Experiment 1, we planned to start with a first batch of 120 participants (i.e., 30 participants in each of our four experimental groups; with half of the participants per group recruited in Geneva and the other half in Austin). We planned to analyze the data of this first batch to assess the evidence in the data for or against the interaction of interest (the interaction between Priority Type and Priority Status). If the Bayes factor for or against the interaction of interest would be lower than 10, a second batch of 120 participants would be tested (i.e., again 30 participants in each the four experimental groups, again with half of the participants per group recruited in Geneva and the other half in Austin). Thus, testing would either stop after the first batch of 120 participants because the Bayes factor of interest reached 10, resulting in a final sample size of 120 participants, or stop after the second batch of 120 participants because the Bayes factor of interest did not reach 10 after the first batch, resulting in a final sample size of 240 participants.

In the first batch, we collected data of 125 participants in total, resulting in 103 usable datasets after performing preregistered performance-based exclusions (see main text). The best model of these data was the full model, and this model was over 150 times better than the model with only the two main effects (i.e., excluding the interaction of interest). As this Bayes factor was higher than 10, data collection stopped and no further data were collected.

**3. Experiments 1 and 3: Breakdown of type of trials**

|  | **No priority signal** | **With priority signal** | |
| --- | --- | --- | --- |
|  |  | **Valid** | **Invalid** |
| **Test-relevance** | 25 | 80 | 20 |
| **Reward** | 25 | 50 | 50 |
| **Refresh** | 25 | 50 | 50 |
| **Test-relevance&Reward** | 25 | 80 | 20 |
| **Total** | **125** | | |

**4. Experiment 2: Breakdown of type of trials**

|  | **No priority signal** | **With priority signal** | |
| --- | --- | --- | --- |
|  |  | **Valid** | **Invalid** |
| **Test-relevance** | 25 | 79 | 20 |
| **Reward** | 25 | 33 | 66 |
| **Refresh** | 25 | 33 | 66 |
| **Test-relevance&Reward** | 25 | 79 | 20 |
| **Total** | **124** | | |

**5. Experiment 1: Additional analyses**

A preregistered exploratory analysis compared prioritized items with baseline trials (without a priority signal). For this, we analyzed recall error using a Bayesian repeated-measures ANOVA, with Priority Type (4 levels: Test-relevance, Reward, Refreshing, Test-relevance&Reward) as a between-subject variable and Priority Status (2 levels: Prioritized, Baseline) as a within-subject variable. In this analysis, Prioritized items were extracted from trials with a priority signal, whereas Baseline items were extracted from trials without a priority signal. The best model was the Priority Status-only model (BF_10_ = 605.75, against the Null). There was strong evidence against including Priority Type (BF_01_ = 26.64), and the full model including the interaction of interest was almost 20 times worse than the best model. Thus, there was also evidence against the memory boost varying between the different prioritization modes when comparing prioritized items to baseline items, rather than to unprioritized items.

Next, to test whether prioritized items, on the one hand, and unprioritized items, on the other hand, show similar memory performance across different prioritization manipulations, we ran two additional, preregistered Bayesian repeated-measures ANOVAs. Prioritized and unprioritized items were both extracted from trials with a priority signal. In the first additional analysis, we tested whether memory performance for prioritized items varies across the different prioritization modes. Recall error for prioritized items was analyzed using a BANOVA with Priority Type (4 levels: Test-relevance, Reward, Refreshing, Test-relevance&Reward) as a between-subject variable. The null model was found to be the best model, with modest evidence against the main effect of Priority Type (BF_01_ = 4.17 against the Null), see Panel A in Figure below. Similarly, in the second additional analysis, we tested whether memory performance for unprioritized items varies across the different prioritization modes, using the same BANOVA on recall error for unprioritized items. The best model was again the null model, with modest evidence against the main effect of Priority Type (BF_01_ = 3.58 against the Null), see Panel B in Figure below. Given that none of these analyses showed evidence for a main effect of Priority Type, we did not carry out any follow-up t-tests.

*Note.* The scale of the Y-axis was chosen to be the same across the two panels, maximizing visibility of relevant information and minimizing the omission of individual data points. With the selected scale, there are 2 omitted data points in Panel B, and none in Panel A.

**6. Experiment 2: Additional analyses**

Like in Experiment 1, a preregistered exploratory analysis compared prioritized items with baseline trials (without a priority signal). For this, we analyzed recall error using a Bayesian repeated-measures ANOVA, with Priority Type (4 levels: Test-relevance, Reward, Refreshing, Test-relevance&Reward) as a between-subject variable and Priority Status (2 levels: Prioritized, Baseline) as a within-subject variable. In this analysis, Prioritized items were extracted from trials with a priority signal, whereas Baseline items were extracted from trials without a priority signal. The best model was the full model, with strong evidence for including the interaction of interest (BF_10_ = 217.15). Thus, there was also evidence for the memory boost varying between the different prioritization modes when comparing prioritized items to baseline items, rather than to unprioritized items.

Next, like in Experiment 1, we ran two additional preregistered Bayesian repeated-measures ANOVAs. In the first, recall error for prioritized items was analyzed using a BANOVA with Priority Type (4 levels: Test-relevance, Reward, Refreshing, Test-relevance&Reward) as a between-subject variable. The best model was found to be the null model, with modest evidence against the main effect of Priority Type (BF_01_ = 3.12 against the Null). This indicates that we should consider memory performance for prioritized items as similar across the different prioritization modes, see Panel A in Figure below. In the second additional analysis, the same BANOVA on recall error for unprioritized items revealed that the best model included the main effect of Priority Type, but the evidence was extremely weak (BF_10_ = 1.54 for Priority Type), see Panel B in Figure below. Therefore, it seems that we should consider memory performance for unprioritized items also as similar across the different prioritization modes.

*Note.* The scale of the Y-axis was chosen to be the same across the two panels, maximizing visibility of relevant information and minimizing the omission of individual data points. With the selected scale, there are 4 omitted data points in Panel B, and none in Panel A.

**7. Experiment 3: Additional analyses**

Like in Experiments 1 and 2, an exploratory analysis compared prioritized items with baseline trials (without a priority signal). For this, we analyzed recall error using a Bayesian repeated-measures ANOVA, with Priority Type (4 levels: Test-relevance, Reward, Refreshing, Test-relevance&Reward) as a between-subject variable and Priority Status (2 levels: Prioritized, Baseline) as a within-subject variable. The best model was the full model including the interaction term, with rather weak evidence for including the interaction (BF_10_ = 2.71). Thus, when comparing prioritized items to baseline items, rather than to unprioritized items, there was still some evidence for the memory boost varying between the different prioritization modes.

Next, we ran two additional Bayesian repeated-measures ANOVAs. In the first, recall error for prioritized items was analyzed using a BANOVA with Priority Type (4 levels: Test-relevance, Reward, Refreshing, Test-relevance&Reward) as a between-subject variable. The best model was the null model, with strong evidence against the main effect of Priority Type (BF_01_ = 13.46 against the Null). Thus, we should consider memory performance for prioritized items similar across different prioritization modes, see Panel A in Figure below. In the second additional analysis, the same BANOVA on recall error for unprioritized items revealed that the best model included the main effect of Priority Type, with strong evidence for the effect (BF_10_ = 22.93), see Panel B in Figure below. Memory performance for unprioritized items varies thus across the different prioritization modes in this experiment.

*Note.* The scale of the Y-axis was chosen to be the same across the two panels, maximizing visibility of relevant information and minimizing the omission of individual data points. With the selected scale, there are 6 omitted data points in Panel B, and none in Panel A.

**8. Additional discussion of costs and benefits with reward-based prioritization**

It is worth noting that, while we consistently observed a benefit for retrospective rewarding across experiments (but not a consistent cost), Hautekiet et al. (2025) reported the opposite pattern, i.e., a consistent cost but not a consistent benefit for reward-based prioritization. Their findings can also be explained through resource reallocation. Specifically, a cost without an associated benefit is consistent with resources being diverted away from unprioritized information, impairing its memory. However, the freed-up resources may have been either left unused for prioritized information or used ineffectively, leading to the absence of a benefit. As such, the findings of Hautekiet et al. (2025) can be accounted for by resources consistently being diverted away from unprioritized information (causing a consistent cost), but only sometimes effectively used to enhance prioritized information (leading to an inconsistent benefit). Thus, our current findings and those of Hautekiet et al. (2025) do not necessarily imply different underlying mechanisms.

**9. Use of AI**

ChatGPT was used for editing purposes. Specifically, it was used to find ways to simplify some complex sentences. Prompts such as “"how can this sentence be simplified?” or “how can this sentence be improved?" were used, but no records of the prompts or conversations were kept.
